# Central precocious puberty secondary to hypothalamic hamartoma

**DOI:** 10.1186/1687-9856-2013-S1-P70

**Published:** 2013-10-03

**Authors:** CG Tay, MY Jalaludin, F Harun

**Affiliations:** 1 Paediatric Endocrine Unit, Department of Paediatrics, Faculty of Medicine, University Malaya, Kuala Lumpur

## Introduction

Central precocious puberty (CPP) presenting at a very young age is likely to have an underlying pathology. One of the pathologies is hypothalamic hamartoma (HH), a non-neoplastic tumour-like lesion located at the floor of the third ventricle, near the tuber cinereum. Two young children with CPP due to HH without gelastic seizures or mental retardation were successfully managed and described in this report.

## Cases

### Case 1

An 18-months-old girl presented with vaginal bleeding. Physical examination showed breast Tanner stage (TS) 4 with pubic hair TS2. Her follicle-stimulating hormone (FSH), luteinizing hormone (LH) and estradiol were in pubertal range (6 IU/L, 8.9 IU/L, 281 pmol/L respectively) with an advanced bone age of 7 years 10 months. Brain magnetic resonance imaging (MRI) showed a round, non-enhancing, isointense lesion at the tuber cinereum just anterior to the mamillary body representing hypothalamic hamartoma. She responded well with intramuscular GnRH analogue with resolution of precocious puberty. She is currently 9 years, remains prepubertal with maintenance dose of GnRH analogue at 4.2mcg/kg/day.

### Case 2

A 20-months-old boy presented with rapid growth. Pubertal staging showed gonads TS2 (testicular volumes of 8mls bilaterally) with pubic hair TS2. His FSH, LH and testosterone were elevated (12 IU/L, 16.5 IU/L and 12.5 nmol/L respectively) with an advanced bone age (BA 3 years). His MRI brain confirmed hypothalamic hamartoma. GnRH analogue at conventional dose failed to correct the excessive growth and stop the progression of pubertal development until a higher dose at 18.5mcg/kg/day was given. He is currently 41/2 years and remains prepubertal.

## Conclusion

CPP secondary to hypothalamic hamartoma usually presents early, before the age of two years. Medical therapy with GnRH analogue is still the first choice of treatment. The dosage of GnRH varied and in patients whom standard dose showed insufficient effects, high dose is recommended. Surgical intervention is reserved only for those who failed medical therapy or for those with intractable seizures.

